# Social Distancing and Outdoor Physical Activity During the COVID-19 Outbreak in South Korea: Implications for Physical Distancing Strategies

**DOI:** 10.1177/1010539520940929

**Published:** 2020-07-15

**Authors:** Sunhee Park, Beomsoo Kim, Jaeil Lee

**Affiliations:** 1 Yonsei University, Seoul, Korea

**Keywords:** social distancing, physical distancing, outdoor activity, COVID-19, Korea

## Abstract

The novel coronavirus disease (COVID-19) outbreak has put the entire world in a pandemic situation. In response, strict screening, quarantine protocols, and contact tracing have been conducted in South Korea. The purpose of this study was to examine effects of social distancing on the Public Bicycle Sharing System (PBSS) during the COVID-19 outbreak. We used the PBSS public dataset of Seoul, South Korea. Difference-in-differences (DID) analysis was used. In the DID approach, the 2 groups are distinguished based on designated year. Cases of PBSS use were observed in 2 time periods: pre- and post-strict social distancing in Seoul, Korea. Average PBSS usage per day doubled during 2019-2020 (30 697 vs 77 996, *P* < .001). Commuters and weekend users increased during the social distancing period in 2020 compared with the same period in 2019. DID analysis showed statistically significant positive effects of high levels of social distancing on PBSS usage, commuters, weekend users, and new subscribers. In conclusion, social distancing during the COVID-19 outbreak increased outdoor physical activity. Meaningful outdoor physical activity during the COVID-19 pandemic can be safe from infection and psychologically stabilized as long as keeping meticulous physical distancing, such as hand hygiene, wearing facial masks, and surface cleaning of public resources.

## Introduction

The World Health Organization (WHO) declared a global pandemic in March 2020 because of the novel coronavirus disease (COVID-19) outbreak.^1^ With no vaccines available, preventive measures and social distancing are the most important precautions.

Preventive measures to mitigate infection spread include good personal hygiene, prioritizing handwashing, cleansing mobile phones and other personal items, practicing respiratory cough etiquette, and wearing masks. Social distancing techniques involve staying at least 6 feet away from others, avoiding crowds, not using public transportation, and following work and quarantine restrictions.^2^ In past pandemics, extreme social distancing measures like isolation and quarantine have been proven to trigger depression and anxiety. People separated from loved ones have a higher risk of developing mental health problems. Thus, reduced social interaction and home quarantine can lead to increased rates of mental health problems.^3^

Physical activity is associated with better quality of life and health outcomes.^4^ Regular physical activity can reduce potential feelings of anxiety, boredom, and depression resulting from social distancing^5^; however, the actual impact of social distancing on individual physical outdoor activity is unknown. This study investigated Public Bicycle Sharing System (PBSS) use as a physical outdoor activity, comparing COVID-19 pandemic exposure and nonexposure and pre– and post–social distancing policy.

## Methods

We used the Seoul, South Korea, PBSS dataset (https://data.seoul.go.kr/dataList/datasetList.do) for a cross-sectional analysis of bicycle use at two points in time: January to March 2019 and January to March 2020. The latter was during the COVID-19 peak, when strict social distancing was imposed in Seoul from March 22. We performed descriptive statistics (independent *t* tests) and difference-in-differences (DID) analyses to examine the effect of social distancing on PBSS use during the COVID-19 outbreak. The DID approach distinguished between two groups: a COVID-19 non-exposure and exposure group based on the designated year. Cases of PBSS use were observed in two time periods, that is, pre– and post–strict social distancing. This is a simple formulation with two groups and two time points. This work complies with American Public Health Association’s ethical practice of public health.

## Results

Average daily PBSS usage in 2020 doubled over that of 2019 (30 697 vs 77 996, *P* < .001) (See supplemental material). Similarly, commuter and weekend users increased significantly. There was a significant difference in time (ie, not only time of day but also day of the week) during which individuals utilized PBSS. DID analysis showed statistically significant positive effects of high levels of social distancing on PBSS usage and number of commuters, weekend users, and new subscribers after controlling for day (Table 1).

**Table 1.** DID Analysis of Effects of Social Distancing on PBSS Use Pre- and Post-COVID-19 Outbreak^a^.

Variables	Overall PBSS		Commuters		Weekend users		New subscribers	
	Coefficient (*P*)	SE	Coefficient (*P*)	SE	Coefficient (*P*)	SE	Coefficient (*P*)	SE
Social distancing	21399.0 (<.000)	3176.1	6792.7 (<.000)	1370.1	15836.0 (<.000)	1915.0	1560.3 (<.000)	283.8
Group	11797.0 (<.000)	1407.3	3575.1 (<.000)	607.1	11268.0 (<.000)	2664.0	316.5 (<.000)	125.8
Social distancing* Group	35503.0 (<.000)	4233.4	9494.4 (<.000)	1826.2	47129.0 (<.000)	7323.0	3657.8 (<.000)	378.3
Day	275.3 (<.000)	30.3	81.9 (<.000)	13.1	1234.5 (<.000)	204.6	23.3 (<.000)	2.7
Adjusted *R*^2^	0.73		0.55		0.76		0.68	

Abbreviations: DID, difference-in-differences; PBSS, Public Bicycle Sharing System; SE, standard error.

a Linear regression model: DID in PBSS use between the COVID-19 nonexposure and exposure groups with high and low social distancing levels. Social distancing = pre- and post–strict social distancing as of March 22, 2020, in Seoul. Sample includes all PBSS use between January and March in 2019 and 2020. Control for the “day” covariate is added.

## Discussion

Our results suggest that social distancing during the COVID-19 outbreak affected PBSS use positively. While prior studies on social distancing in health care have highlighted important preventive behaviors during previous pandemics, this study demonstrates the additional impact of social distancing on outdoor individual physical activity.

### Physically Safe PBSS Use During Social Distancing

PBSS use has been rising annually as a public transportation alternative and for leisure.^6^ In this study, people voluntarily forsook convenient public transportation to use personal transportation instead, specifically PBSS, to follow social distancing instructions and prevent the spread of COVID-19 in commuters. Since viruses can be spread via public resources’ surfaces, when using PBSS, individual physical distancing practices, like hand hygiene, surface cleaning, and wearing masks, are needed. To stop the spread of infection via PBSS, it is necessary for PBSS authorities to provide these hand sanitizers or sprays around the PBSS stops.

### PBSS Use for Psychological Health

Physical exercise has been shown to effectively reduce psychological impacts of isolation like boredom, loneliness, and depression.^5^ Increased weekend PBSS usage illustrates the rise of individual physical outdoor activities over group activities or staying home during social distancing. For people suffering from anxiety and stress after more than 3 months of social distancing, individual outdoor physical activities such as bicycling could help relieve psychological depressive symptoms or boredom.

## Public Health Implications

Meaningful outdoor physical activities during the COVID-19 pandemic can be safe from infection and psychologically stabilizing if meticulous physical distancing is maintained. The WHO recently recommended the term “physical distancing” rather than “social distancing” to make people feel less isolated.^1^ It is important to note that during outdoor activities, physical distancing measures, including hand hygiene, wearing masks, and surface cleaning of public resources and electronic devices, are of top priority.

## Supplemental Material

Supplementary_Table – Supplemental material for Social Distancing and Outdoor Physical Activity During the COVID-19 Outbreak in South Korea: Implications for Physical Distancing Strategies Click here for additional data file. Supplemental material, Supplementary_Table for Social Distancing and Outdoor Physical Activity During the COVID-19 Outbreak in South Korea: Implications for Physical Distancing Strategies by Sunhee Park, Beomsoo Kim and Jaeil Lee in Asia Pacific Journal of Public Health
