# Supplementary material for: Social Distancing and Outdoor Physical Activity During the COVID-19 Outbreak in South Korea: Implications for Physical Distancing Strategies
Source: Asia Pac J Public Health. 2020 Jul 15;32(6-7):360–2. doi: 10.1177/1010539520940929 (PMC7364329; doi:10.1177/1010539520940929)
Supplement: Supplementary_Table – Supplemental material for Social Distancing and Outdoor Physical Activity During the COVID-19 Outbreak in South Korea: Implications for Physical Distancing Strategies [file Supplementary_Table.pdf]

Supplementary Table. PBSS use from January to March 2019 and 2020 of COVID-19 exposure and non-exposure groups

Type	Social distancing implementation	Group by year		t (p^a)
		2019	2020	
Overall PBSS	Pre	18934(±7041)	30868(± 13809)	2.13(0.04)
	Post	30696(±9367)	77996(±8619)	6.52(<0.001)
Commuters	Pre	6128.2(±2928)	9744(±4958)	5.64(<0.001)
	Post	10054(±4977)	23124(±6026)	4.86(<0.001)
Weekend users	Pre	17015(±8337)	28900(± 15108)	3.29(<0.01)
	Post	21747(±7084)	79520(±2896)	14.75(<0.001)
New subscribers	Pre	763(±587)	1091(±1101)	2.36(0.02)
	Post	1509(±596)	5483(±1874)	5.87(<0.001)

2019=non-exposure group; 2020=exposure group

Pre-social distancing = Form January 1st~ March 21th.; Post-social distancing=March 22th~ March 31th
